# *Elaeagnus latifolia* Fruit Extract Ameliorates High-Fat Diet-Induced Obesity in Mice and Alleviates Macrophage-Induced Inflammation in Adipocytes In Vitro

**DOI:** 10.3390/antiox13121485

**Published:** 2024-12-05

**Authors:** Narongsuk Munkong, Nattanida Jantarach, Bhornprom Yoysungnoen, Piyanuch Lonan, Jiradej Makjaroen, Phorutai Pearngam, Sarinya Kumpunya, Kingkarnonk Ruxsanawet, Saharat Nanthawong, Poorichaya Somparn, Arthid Thim-Uam

**Affiliations:** 1 Department of Pathology, School of Medicine, University of Phayao, Phayao 56000, Thailand; narongsuk.mu@up.ac.th; 2 Applied Thai Traditional Medicine Program, School of Public Health, University of Phayao, Phayao 56000, Thailand; nattanida.ja@up.ac.th (N.J.); kingkarnonk.ru@up.ac.th (K.R.); 3 Division of Physiology, Department of Preclinical Science, Faculty of Medicine, Thammasat University, Pathum Thani 12120, Thailand; pornprom@tu.ac.th; 4 Traditional Chinese Medicine Program, School of Public Health, University of Phayao, Phayao 56000, Thailand; piyanuch.lo@up.ac.th; 5 Department of Transfusion Medicine and Clinical Microbiology, Faculty of Allied Health Sciences, Chulalongkorn University, Bangkok 10330, Thailand; jiradej.ma@chula.ac.th; 6 Center of Excellence in Systems Biology, Faculty of Medicine, Chulalongkorn University, Bangkok 10330, Thailand; saharat.m@chula.ac.th (S.N.); poorichaya.s@chula.ac.th (P.S.); 7 International College, Mahidol University, Nakhon Pathom 73170, Thailand; phorutai.pea@mahidol.edu; 8 Division of Infectious Diseases, Department of Medicine, Faculty of Medicine, Chulalongkorn University and King Chulalongkorn Memorial Hospital, Thai Red Cross Society, Bangkok 10330, Thailand; ple_poy@hotmail.com; 9 Division of Biochemistry, School of Medical Sciences, University of Phayao, Phayao 56000, Thailand

**Keywords:** *Elaeagnus latifolia*, obesity, inflammation, macrophages infiltration, energy-producing pathways

## Abstract

*Elaeagnus latifolia* (EL) is a wild fruit known for containing several health-promoting compounds. This study aimed to evaluate the effects of EL fruit extract on high-fat diet (HFD)-induced obesity and lipopolysaccharide (LPS)-activated macrophages. Mice fed an HFD and given EL fruit extract for 10 weeks exhibited significantly lower body weight, reduced lipid accumulation, diminished oxidative stress in adipocytes, and decreased macrophage infiltration compared to those not receiving the EL extract. Moreover, the EL fruit extract activated the transcription factors *Pparg* and *Cebpa*, initiating adipogenesis and modulating the expression of NF-κB/Nrf-2-induced target genes. This resulted in smaller adipocyte size, reduced inflammation, and less oxidative stress in HFD-fed mice. In vitro, the EL extract induced a shift in macrophage phenotype from M1 to M2, reduced IκBα/NF-κB phosphorylation, and effectively decreased energy production in macrophages by downregulating the expression of several proteins involved in glycolysis and the tricarboxylic acid cycle. This mechanistic study suggests that administering EL fruit extract could be an effective strategy for managing obesity and its associated pathologies.

## 1. Introduction

Obesity is primarily caused by pathological lipid accumulation in various tissues and organs, particularly in the visceral or abdominal region, including white adipose tissue (WAT), a condition known as abdominal obesity [1]. This form of obesity is a significant contributor to a range of serious non-communicable diseases, including diabetes mellitus, metabolic dysfunction-associated fatty liver disease (MAFLD), cardiovascular diseases, hypertension, and renal diseases [2,3,4]. Impaired adipogenesis, the process of adipocyte differentiation, has been suggested as a key pathological mechanism in obesity [5]. This is due to the production of abnormal adipocytes that lose their normal functions of lipid storage and small size in response to high-energy insult. As a result, pathological adipocytes accumulate, and the adipose tissue is unable to expand through adipocyte hypertrophy (increase in size). Moreover, the release of lipids or free fatty acids (FFAs) into non-adipose tissues, a phenomenon known as ectopic lipid accumulation, occurs. This differs from healthy WAT remodeling, which is characterized by a large number of small, functionally normal adipocytes in metabolically healthy obesity [5]. Therefore, it is hypothesized that increasing the number of mature adipocytes (hyperplasia) with lipid storage capacity, which facilitates caloric storage via adipogenesis, could be a beneficial physiological adaptation to prevent the accumulation of pathological adipocytes and excess fat in other organs.

Adipogenesis is initiated by the activation of adipogenic transcription factors such as peroxisome proliferator-activated receptor-gamma (PPARG) and CCAAT/enhancer-binding protein-alpha (CEBPA). Dysregulation of these factors can impair adipogenesis. Additionally, adipose tissue hypoxia, which occurs following adipose tissue expansion, activates the hypoxia-inducible factor-1α (HIF1A) cascade, impairing adipocyte function and adipogenesis [6]. Unhealthy WAT remodeling is also characterized by increased inflammation and oxidative stress [5]. Enlarged adipocytes secrete chemokines that recruit immune cells, primarily macrophages, to surround adipocytes, forming histological crown-like structures (CLSs). This process amplifies the hyperinflammatory state by activating pro-inflammatory regulators such as nuclear factor-kappa B (NF-κB), which in turn upregulates the expression of inflammatory genes and overwhelms anti-inflammatory regulators such as interleukin 10 (IL10) [7]. Additionally, oxidative stress resulting from reactive oxygen species (ROS) production can overwhelm antioxidant regulation, particularly the nuclear factor erythroid 2-related factor 2 (NFE2L2/NRF2)-mediated antioxidant gene expression [8]. Both inflammation and oxidative stress contribute to metabolic complications, including disrupted adipogenesis, insulin resistance, and dyslipidemia [9]. Therefore, strategies aimed at reversing unhealthy WAT remodeling, improving adipogenesis to produce normal adipocytes, preventing adipocyte hypertrophy, and inhibiting inflammation and oxidative stress could be promising approaches to combating obesity and metabolic syndrome.

Consuming foods or natural products rich in bioactive compounds and low in energy density, such as fruits and vegetables, has been recommended as a strategy for controlling obesity and its related complications [10]. Wild edible fruits and vegetables from various regions are known to contain diverse bioactive compounds, such as phenolics, which have potential metabolic-regulating, antioxidant, and anti-inflammatory properties [11]. *Elaeagnus latifolia* Linn. (EL), a wild fruit from the Elaeagnaceae family, is found in Asian countries such as Thailand, Vietnam, India, and Sri Lanka. EL fruit contains several beneficial health constituents, including phenolic compounds (e.g., quercetin, catechin, and rutin), vitamins, minerals, essential fatty acids, and carbohydrates [12]. In vitro experiments have shown that EL fruit extracts possess antioxidant, DNA-protective, and antimicrobial activities, which may be attributed to their bioactive constituents, particularly polyphenolic flavonoids, carotenoids, and ascorbic acid [11,12,13]. A previous in vivo study reported that EL fruit extract, rich in phenolic and flavonoid phytochemicals, exhibited hepatoprotective effects in a model of N-acetyl-para-aminophenol overdose. This protection was attributed to enhanced antioxidant activity, reduced inflammation, and decreased hepatic lipid accumulation [14].

Extracts containing polyphenols and flavonoids from other fruits, including compounds such as quercetin, catechin, and rutin, have shown beneficial effects on parameters involved in metabolic dysfunction. These include reductions in visceral adipose tissue, lipid accumulation, and indicators of oxidative stress and inflammation in animal models of obesity, as well as in adipocytes and macrophages [15,16]. Although detailed studies on the pharmacological effects of EL fruit extract and its bioactive compounds are ongoing, the results so far suggest that these compounds may help prevent inflammation, oxidative stress, and metabolic alterations. However, the effects of EL fruit extract on the molecular pathogenesis of abdominal obesity, the inflammatory immune response, and oxidative stress in chronic metabolic disease models, as well as its role in macrophages, have not yet been fully verified. Therefore, the present study aimed to investigate the anti-obesity, anti-inflammatory, and antioxidant properties of EL fruit extract using a typical in vivo obesity model with HFD-induced mice and an in vitro model with lipopolysaccharide (LPS)-induced RAW 264.7 macrophages. The study also examined the in vitro free radical-scavenging activities and bioactive compound contents of EL fruit extract.

## 2. Materials and Methods

### 2.1. Preparation of Aqueous Extracts of EL Fruits

A voucher specimen of EL from Mon-Ma-Meun Thailand Permaculture Farm, Phayao Province, Thailand (specimen number 2264), was deposited at the herbarium of the Center of Excellence in Agricultural Innovation for Graduate Entrepreneur, Maejo University, Chiang Mai Province, Thailand. The edible parts (flesh and pulp) of EL fruits were collected, blended, and subjected to a water-based extraction process to minimize the risks associated with toxic solvent residues, following methods outlined in previous publications [17]. Briefly, 1 kg of the blended edible parts was boiled in 2 L of distilled water at 80 °C for one hour. The mixture was then filtered through a cotton layer at room temperature using a vacuum filtration system consisting of a Büchner funnel, filter flask, and pump. The filtrate was subsequently lyophilized to achieve dryness. The resulting extract was weighed to calculate the percentage yield and stored at −80 °C until used for further in vitro and in vivo assays.

### 2.2. Determination of Total Phytochemical Contents

The total flavonoid content (TFC) and total phenolic content (TPC) of aqueous EL fruit extracts were determined using colorimetric methods, as described in previous publications [18,19]. Briefly, to measure the TFC, the EL extract was mixed with 5% sodium nitrite (NaNO_2_) and 10% aluminum chloride (AlCl_3_) and allowed to react for five minutes. Next, 1 M sodium hydroxide (NaOH) was added to the mixture. The resulting solution was then measured at 510 nm using a UV-Vis spectrophotometer (Thermo Fisher Scientific, Waltham, MA, USA), and the TFC was expressed as mg catechin per gram of extract. To determine the TPC, the EL fruit extract was mixed with 10% Folin–Ciocalteu solution and 7.5% sodium carbonate (Na_2_CO_3_), and the reaction was carried out in the dark at room temperature for 15 min. The absorbance of the reaction mixture was then measured at 765 nm using a UV-Vis spectrophotometer (Thermo Fisher Scientific, Waltham, MA, USA). The TPC was expressed as mg of gallic acid equivalent (GAE) per gram of extract.

### 2.3. Determination of In Vitro Antioxidant Activity

The in vitro antioxidant activity of the EL aqueous extract was evaluated using the 2,2-diphenyl-1-picrylhydrazyl (DPPH) and 2,2′-azino-bis(3-ethylbenzothiazoline-6-sulfonic acid) (ABTS) assays (Fluka, Buchs, Switzerland), following the manufacturer’s instructions. Briefly, a 150 mM DPPH solution was mixed with varying concentrations of the EL fruit extract and allowed to react for 10 s. Similarly, a 7 mM ABTS solution was mixed with different concentrations of the EL fruit extract for 10 s. The reaction mixtures were then measured using a UV-Vis spectrophotometer (Thermo Fisher Scientific, Waltham, MA, USA) at 765 nm for the DPPH assay and 734 nm for the ABTS assay. Trolox was used as a positive control. The results were expressed as the half-maximal inhibitory concentration (IC_50_) against DPPH and ABTS radicals.

### 2.4. Characterization of Bioactive Contents

The bioactive compounds in the aqueous EL fruit extract were identified using liquid chromatography–mass spectrometry (LC-MS) at the Center of Excellence for Medicinal Plants and Thai Traditional Medicine, Mae Fah Luang University, Chiang Rai, Thailand. Briefly, 10 mg of dried EL fruit powder was dissolved in LC-grade water and then adjusted to a final concentration of 1 mg/mL using LC-grade methanol. The solution was filtered through a 0.22 µm PTFE syringe filter (Merck, Darmstadt, Germany) before being injected into the system. Separation was performed using an Agilent 1290 Infinity LC instrument coupled with an Agilent 6540 Series QTOF MS, equipped with an electrospray ionization source and a diode-array detector. An Agilent Poroshell 120 EC-18C column (4.6 × 150 mm, 2.7 µm) was used for the separation at 35 °C. The flow rate was set to 200 µL/min, and 20 µL of the sample was injected. The mobile phase consisted of LC-grade water (with 0.1% formic acid) and acetonitrile (with 0.1% formic acid). Data analysis was conducted using Agilent MassHunter Workstation software (version B.08.00; Agilent, Santa Clara, CA, USA), and bioactive compounds were identified using the Personal Compound Database and Library.

### 2.5. Animal Study

**Figure 1:**
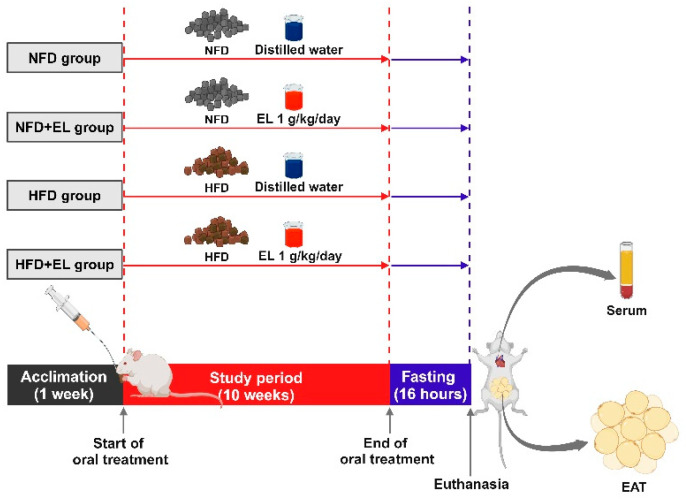
Schematic illustration of animal experiment. The mice were divided into four groups, and EL was fed to HFD-fed over 10 weeks. This representative figure was created with BioRender.com (accessed on 19 November 2024).

The animal protocol (number 1-013-65) was approved by the Laboratory Animal Use Committee at the Laboratory Animal Research Center, University of Phayao, Phayao, Thailand, in accordance with the guidelines set by the US National Institutes of Health (NIH). Four-week-old male ICR mice (Nomura Siam International Co., Ltd., Bangkok, Thailand) were housed in the animal experimental room at 22 ± 3 °C with 50 ± 10% relative humidity and a 12/12 h light/dark cycle for one week to allow acclimation. Following this period, the mice were divided into four groups (n = 6/group; see Figure 1): (1) mice fed a normal fat diet (NFD) and co-administered sterile distilled water, (2) mice fed an NFD and co-administered the EL fruit extract at 1 g/kg/day (NFD + EL), (3) mice fed a high-fat diet (HFD) and co-administered sterile distilled water, and (4) mice fed a HFD and co-administered the EL fruit extract at 1 g/kg/day (HFD + EL). The NFD, which contained 13% kcal from fat (energy density = 3.04 kcal/g), was purchased from the National Laboratory Animal Center at Mahidol University (Nakhon Pathom, Thailand; number 082G [082]), while the HFD, which contained 60% kcal from fat (energy density = 5.97 kcal/g), was prepared from ingredients typically used in human diets, including pork lard, pork liver, margarine, sugar, wheat flour, and whole hen eggs (Makro, Phayao, Thailand).

The body weight (BW), food intake, and energy intake of the mice were recorded weekly. The mice were administered either sterile distilled water or EL fruit extract dissolved in sterile distilled water via an oral feeding tube daily for 10 weeks. The preparation of the HFD, the timing of animal treatments, and the dose of the EL fruit extract were based on a previous animal study with some modifications [20]. At the end of the study, the mice were fasted for 15 h overnight and anesthetized with carbon dioxide (CO_2_), and blood samples were collected via cardiac puncture. Following the blood collection, the abdominal cavity was opened, and the epididymal adipose tissue (EAT), a major depot of abdominal white adipose tissue (WAT), was weighed and collected for further histopathological and biochemical analyses.

### 2.6. Histological Examination of EAT

Immediately after collection, the EAT specimens were fixed in 10% formalin, paraffin-embedded, and sectioned into 5 μm thick slices. These sections were then stained with hematoxylin and eosin (H&E) (C.V. Laboratories, Bangkok, Thailand) and imaged using a BX53 light microscope equipped with an SC180 digital camera (Olympus Corporation, Tokyo, Japan). The size of 100 adipocytes from each mouse (n = 3/group) was measured in 10 random fields at ×200 magnification using Olympus cellSens software (version 4.2). The formation of crown-like structures (CLSs) was also assessed in each ×200 field, and the number of CLSs per field was manually recorded.

Macrophage infiltration in EAT was analyzed using immunohistochemistry. Briefly, the EAT sections (5 μm) were incubated with an anti-F4/80 antibody (Invitrogen, Waltham, MA, USA; dilution 1:200) at 23 °C for 1 h. Following this, the sections were incubated with a biotinylated goat anti-rabbit secondary antibody (Cell Signaling, Danvers, MA, USA; dilution 1:200) at 23 °C for 30 min. The staining was visualized using a BX53 light microscope (Olympus Corporation, Tokyo, Japan), and the sections were examined in 10 random fields at ×400 magnification, as previously described [21].

### 2.7. Measurement of Lipids in EAT

Briefly, 50 mg of EAT was homogenized in 1000 µL of a methanol–chloroform (2:1, *v*/*v*) solution using an electronic tissue homogenizer. The homogenates were then centrifuged at 10,000 rpm for 10 min at 4 °C. Following the respective kit protocols, the total cholesterol and triglyceride (TG) levels were quantified using lipid assay kits (Erba Diagnostics Mannheim GmbH, Mannheim, Germany), and the free fatty acid (FFA) levels in EAT were measured using an FFA assay kit (Fujifilm Wako Pure Chemical Corporation, Osaka, Japan). The lipid levels in EAT were expressed as μg of lipid per gram of EAT.

### 2.8. Measurement of Oxidative Stress

To measure oxidative stress in the tissue, EAT was homogenized in 1000 µL of 0.05 M phosphate-buffered saline (PBS) at pH 7.4. After centrifugation at 12,000 rpm for 15 min at 4 °C, the supernatant was collected and incubated with 200 µM 2′,7′-dichlorodihydrofluorescein diacetate (DCF-DA; Sigma-Aldrich, Saint Louis, MO, USA) in the dark. Reactive oxygen species (ROS) production was assessed using a fluorescent microplate reader, with the results reported as a percentage of the NFD group.

To investigate the effects of EL fruit extract on ROS production in RAW 264.7 macrophages, 2 × 10^4^ cells were seeded in 96-well plates and pretreated with various concentrations of EL fruit extract for 1 h. Cells were then activated with or without 1 µg/mL of LPS at 37 °C with 5% CO_2_ for 24 h. Afterward, the cells were washed twice with warm PBS, and 100 µL of warm PBS containing 40 µM DCF-DA was added. The cells were incubated at 37 °C with 5% CO_2_ for 25 min. Fluorescence signals were measured at 485 nm (excitation) and 535 nm (emission) using a fluorescent microplate reader (Thermo Fisher Scientific, Waltham, MA, USA), and a representative fluorescent image was captured with a fluorescence microscope. The data are expressed as the percentage of untreated cells.

The production of malondialdehyde (MDA), a lipid peroxidation indicator, in EAT was measured by mixing the supernatant with 10% thiobarbituric acid (Sigma-Aldrich, Saint Louis, MO, USA) and 0.67% trichloroacetic acid (Merck, Darmstadt, Germany) before boiling the mixture for 15 min at 100 °C. The protein concentration in the supernatant was then determined using the Bradford Protein Assay kit (Bio-Rad, Hercules, CA, USA) according to the manufacturer’s instructions. The results were expressed as nmol of MDA per mg protein in EAT. The oxidative stress evaluation protocols were performed as previously described [14].

### 2.9. Cell Culture

The mouse macrophages RAW267.4 (ATCC number; TIB-71) were purchased from Biomedia Thailand (ATCC distributor) and cultured in complete medium (high-glucose Dulbecco’s modified Eagle medium with 10% fetal bovine serum (HyClone, Logan, UT, USA) and antibiotics (Gibco–Thermo Fisher Scientific, Waltham, MA, USA) at 37 °C with 5% CO_2_. Before all subsequent experiments, the macrophages were counted, seeded, and cultured in culture plates for 24 h.

### 2.10. Cell Viability

The cytotoxic effect of the aqueous EL fruit extract on RAW 264.7 macrophages was assessed using the 3-(4,5-Dimethylthiazol-2-yl)-5-(3-carboxymethoxyphenyl)-2-(4-sulfophenyl)-2H-tetrazolium inner salt (MTS) assay, following the manufacturer’s protocol (Promega, Madison, WI, USA). Briefly, RAW 264.7 cells (2 × 10^4^ cells/well) were cultured in 96-well plates and treated with 200 µL of the EL fruit extract at concentrations ranging from 100 to 500 µg/mL for 24 or 48 h. After treatment, the cells were washed twice with complete medium and resuspended in 100 µL of fresh complete medium. Then, 20 µL of MTS solution was added, and the cells were incubated at 37 °C with 5% CO_2_ for two hours. Cell viability was determined by measuring absorbance at 490 nm using a Cytation 5 Microplate Reader (Thermo Fisher Scientific, Waltham, MA, USA). The results were expressed as cell viability relative to untreated cells.

### 2.11. Measurement of Nitric Oxide (NO) Production

As previously described, RAW 264.7 macrophages were cultured and treated with the EL fruit extract, with or without lipopolysaccharide (LPS). After treatment, the culture supernatant was collected, and NO levels were quantified using the Griess reagent assay. Briefly, equal volumes (1:1 ratio) of 1% sulfanilamide (Sigma-Aldrich, Saint Louis, MO, USA) in 5% phosphoric acid (Sigma-Aldrich, Saint Louis, MO, USA) and 0.1% N-(1-naphthyl) ethylenediamine dihydrochloride (Sigma-Aldrich, Saint Louis, MO, USA) in deionized water were added to the culture supernatant (100 µL each). The mixture was incubated in the dark at room temperature for 10 min. The absorbance was measured at 540 nm using a Cytation 5 Microplate Reader (Thermo Fisher Scientific, Waltham, MA, USA). The results are presented as the percentage relative to the untreated cells.

### 2.12. Flow Cytometer Analysis

To characterize the M1/M2 macrophage phenotypes, flow cytometry analysis was performed as previously described [22]. Briefly, RAW 264.7 macrophages (1 × 10^6^) were cultured and pretreated with 500 µg/mL of the EL extract for 1 h, then stimulated with or without 1 µg/mL of LPS for 24 h in a six-well plate. The activated macrophages were stained with PE-anti-mouse F4/80 (cat: 123110; clone: BM8; BioLegen, San Diego, CA, USA) and analyzed for M1-like markers using PE-Cy7-anti-mouse CD80 (cat: 104734; clone: 16-10A1; BioLegend, San Diego, CA, USA) and FITC-anti-mouse I-Ab (cat: 116405; clone: AF6-120.1; BioLegend). M2-like macrophages were identified using CD206 (cat: 141708; clone: C068C2; BioLegend). Cell viability was assessed using the Viability Dye eFluor 780 (Thermo Fisher Scientific, Waltham, MA, USA) to exclude dead cells. The mature macrophages were then analyzed using a BD LSR-II Flow Cytometer (BD Biosciences, San Diego, CA, USA), and the data were processed using FlowJo software (version 10).

### 2.13. Measurement of Cytokines

Inflammatory cytokine levels in the culture supernatant and serum were measured using the LEGENDplex Mouse Inflammation Panel 13-plex kit (BioLegend), following the manufacturer’s instructions. The coated beads were analyzed on a BD LSR-II Flow Cytometer (BD Biosciences), and the data were processed using LEGENDplex Data Analysis Software Version 2024-06-15.

### 2.14. Genes Expression Analysis

Total RNA was extracted from EAT and macrophages using TRIzol reagent (Invitrogen, Waltham, MA, USA) and purified with the RNeasy Mini kit (Qiagen, Hilden, Germany), following the manufacturer’s instructions. Complementary DNA (cDNA) was synthesized from 1 µg of the extracted RNA using iScript Supermix (Bio-Rad, Hercules, CA, USA). mRNA levels of target genes were quantified using quantitative polymerase chain reaction (qPCR) with TaqMan assays (Applied Biosystems™, Thermo Fisher Scientific, Waltham, MA, USA), according to the manufacturer’s instructions. Relative mRNA levels were normalized to Gapdh expression. The results are presented as fold induction, calculated using the 2^−ΔΔCt^ formula. The IDs for each TaqMan gene expression assay are provided in Supplementary Table S1.

### 2.15. Protein Expression Analysis

RAW 264.7 macrophages (1 × 10^6^ cells) were cultured and pretreated with 500 µg/mL of the EL fruit extract for 1 h. Afterward, the cells were stimulated with or without 1 µg/mL of LPS for 60 min. The cells were then lysed on ice using a lysis buffer containing 10 mM of Tris-HCl, 1% Triton-X100, 150 mM of NaCl, 5 mM of EDTA (pH 8), protease inhibitor cocktail, and phosphatase inhibitors (Thermo Fisher Scientific, Waltham, MA, USA). Total protein was collected from the cell lysates by centrifugation (13,000× *g* for 15 min at 4 °C) and quantified using the bicinchoninic acid assay (Thermo Fisher Scientific, Waltham, MA, USA). Subsequently, 25 µg of total protein was separated on a 12.5% polyacrylamide gel and transferred to a nitrocellulose membrane. The membrane was then incubated overnight at 4 °C with primary antibodies against the following proteins: NF-κB subunit v-rel reticuloendotheliosis viral oncogene homolog A (RELA/p65; clone: D14E12; 1:1000), phosphorylated (Ser536) NF-κB p65 (clone: 93H1; 1:1000), IκBα (clone: 44D4; 1:1000), phosphorylated IκBα (Ser32) (clone: 14D4; 1:1000), and ACTB (clone: 13E5; 1:2500). After washing with 1× Tris-buffered saline containing 0.1% Tween 20, the membrane was incubated with a fluorescent secondary antibody (IRDye^®^ 680 anti-rabbit IgG; 1:10,000; LI-COR, Lincoln, NE, USA) for 1 h at room temperature in the dark. The bands of target proteins were detected using the ODYSSEY CLx system (LI-COR, Lincoln, NE, USA), and the intensity of the bands was quantified using ODYSSEY CLx software version 1.0.11, with normalization to a housekeeping protein.

### 2.16. Sample Preparation and Proteomic Analysis

LPS-stimulated RAW 264.7 macrophages, with and without EL treatment, were suspended in 8 M urea (Merck, Darmstadt, Germany) prepared in 100 mM of triethylammonium bicarbonate buffer (Sigma-Aldrich, Saint Louis, MO, USA) containing a protease inhibitor cocktail (Thermo Fisher Scientific, Waltham, MA, USA). The cells were then lysed via ultrasonication for one minute, with nine-second pulses separated by two seconds. After centrifugation, the protein concentrations were determined, and 100 µg of each sample was reduced with dithiothreitol, alkylated with 2-iodoacetamide, and digested with trypsin (1:50 *w*/*w*). The tryptic digests from untreated, LPS-treated, and LPS + EL-treated cells were labeled with light, medium, and heavy isotopes of dimethyl, respectively. The labeled samples were fractionated using a High pH Reversed-Phase Peptide Fractionation Kit (Thermo Fisher Scientific, Waltham, MA, USA) with minor modifications. Each fraction was then resuspended in 0.1% formic acid (Sigma-Aldrich, Saint Louis, MO, USA) and injected into an EASY-nLC1000 LC-MS system coupled to a Q-Exactive Orbitrap Plus MS with a nano-electrospray ion source (Thermo Fisher Scientific, Waltham, MA, USA). Peptides were eluted using a gradient of 5–40% acetonitrile in 0.1% formic acid. The mass spectrometry (MS) analysis involved a full scan followed by 10 MS2 scans. The resulting data were processed using Proteome Discoverer™ (version 2.1) and SEQUEST-HT software. The MS data were searched against the Mouse Swiss-Prot database (17,185 proteins; October 2023) and the common protein contaminants from The Global Proteome Machine’s Repository of Adventitious Proteins (www.thegpm.org/crap/ (25 October 2023)). The Precursor Ions Quantifier node in Proteome Discoverer was used to calculate the relative MS signal intensities of the dimethyl-labeled peptides. Control channels were used as denominators to calculate abundance ratios of LPS/Control and LPS + EL/LPS. The normalized ratios were logarithmically transformed (Log_2_) to assess differential protein levels. The mean and standard deviation of fold changes in protein abundance were calculated across all three biological replicates. Differentially regulated proteins were identified using unpaired *t*-tests, with a *p*-value of <0.05 considered statistically significant. The mass spectrometry proteomics data are available via ProteomeXchange under the identifier PXD046383.

### 2.17. Statistical Analysis

Statistical analyses were performed using GraphPad Prism (version 8.0; GraphPad Software, Inc., Boston, MA, USA). Data are presented as the mean ± standard error of the mean (SEM). Comparisons across multiple groups were performed using a one-way analysis of variance (ANOVA), while differences between two groups in the proteomics analysis were assessed using a two-tailed Mann–Whitney U test. A *p*-value of <0.05 was considered statistically significant.

## 3. Results

### 3.1. Total Bioactive Compounds and Antioxidant Activity of the EL Fruit Extract

On average, 9.86 g of EL fruit extract was obtained from 100 g of EL fruit. The phytochemical analysis of the aqueous EL fruit extract revealed a total phenolic content (TPC) of 13.88 ± 0.41 mg GAE/g and a total flavonoid content (TFC) of 2.79 ± 0.10 mg catechin/g. The IC_50_ values of the extract against DPPH and ABTS radicals were also determined (Table 1). Additionally, bioactive compounds in the EL fruit extract were identified using LC-MS/MS, which analyzes compounds based on their charge. A total of 29 compounds were detected, as shown in Table 2.

### 3.2. EL Fruit Extract Improves Obesity in HFD-Fed Mice

**Figure 2:**
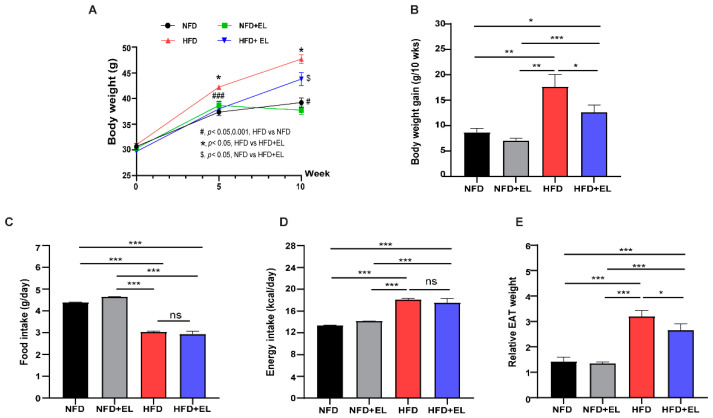
Effects of EL fruit extract on obesity in HFD-fed mice. Treatment with the EL fruit extract reduced (**A**) BW, (**B**) body weight gain (BWG), (**C**) food intake (FI), (**D**) energy intake (EI), and (**E**) relative epididymal white adipose tissue (EAT) weight (n = 6/group). Data are presented as the mean ± standard error (SEM). Statistical significance is exhibited as *, *p* < 0.05; **, *p* < 0.01; ***, *p* < 0.001; #, *p* < 0.05; ###, *p* < 0.001; ns, nonsignificant.

**Figure 3:**
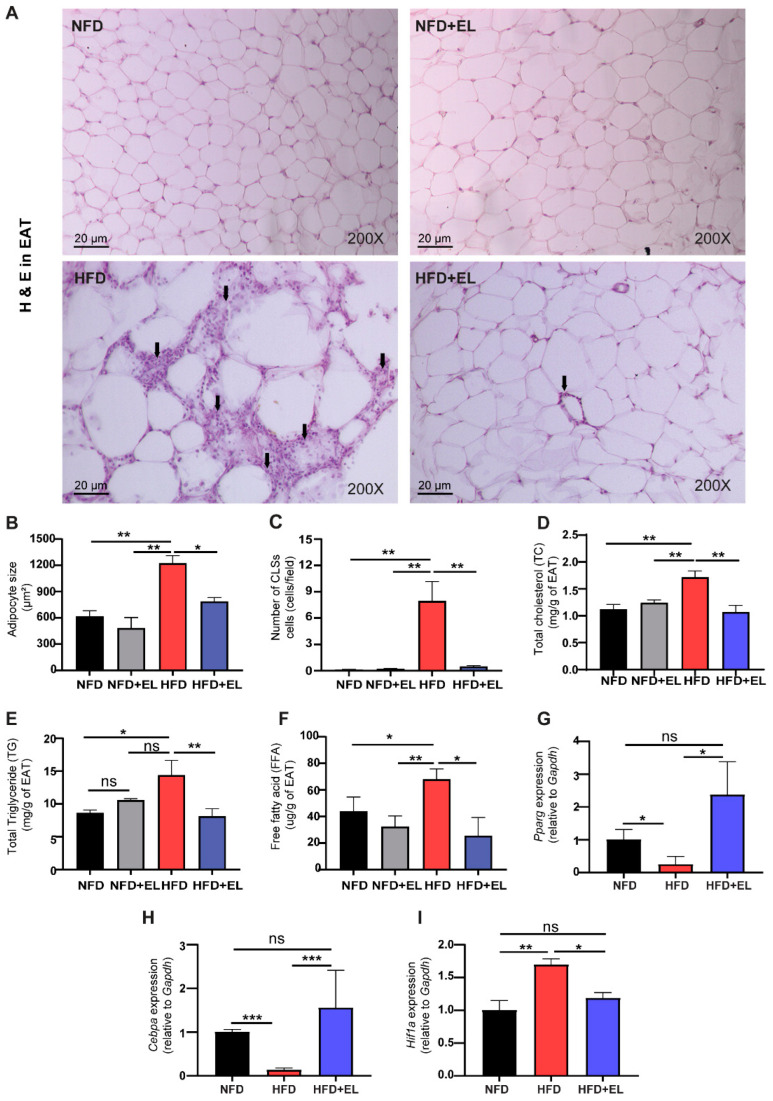
Effects of EL fruit extract on adipogenesis in HFD-diet mice. (**A**) The representative morphology of EAT was visualized through hematoxylin and eosin (H&E) staining at 200× magnification; arrows indicate crown-like structure (CLS) surrounding the adipocytes. (**B**) Adipocyte size and (**C**) number of CLSs were investigated (n = 6/group). The lipid profiles of (**D**) total cholesterol, (**E**) total triglycerides (TGs), and (**F**) free fatty acids (FFAs). The mRNA levels of adipogenesis-related genes (**G**) *Pparg*, (**H**) *Cebpa*, and (**I**) *Hif1a* were demonstrated (n = 6/group). Results are presented as the mean ± standard error (SEM). Statistical significance is exhibited as *, *p* < 0.05; **, *p* < 0.01; ***, *p* < 0.001; ns, nonsignificant.

For our study, mice were divided into four groups, as shown in Figure 1. BWs were significantly higher in HFD-fed mice not treated with the EL fruit extract compared to HFD-fed mice that received the extract after 10 weeks (Figure 2A,B). However, food and energy intake did not significantly differ between these two groups (Figure 2C,D). Additionally, EAT weights were significantly higher in HFD-fed mice not receiving the EL fruit extract compared to those that received it (Figure 2E). Adipocytes were also significantly larger in HFD-fed mice not given the EL fruit extract than in those given the extract (Figure 3A,B). Furthermore, the number of CLS was significantly lower in HFD-fed mice that received the EL fruit extract compared to those that did not (Figure 3C). The lipid profiles in the EAT were also assessed. The data revealed that total cholesterol, TG, and FFA levels were significantly lower in HFD-fed mice treated with the EL fruit extract compared to those not receiving the extract (Figure 3D–F). Overall, general parameters such as body weight, food intake, and energy intake, as well as weight, histology, and lipid levels of adipose tissues, were normal in the NFD + EL group, similar to the NFD group. Our data suggest that treatment with EL fruit extract is safe under normal health conditions. Therefore, this study investigated the molecular mechanisms of EL fruit extract in the context of HFD-induced obesity by comparing the HFD + EL group with the NFD and HFD groups.

### 3.3. EL Fruit Extract Reduces the Adipocyte Size by Increasing Adipogenesis

To assess adipocyte development, the expression of adipogenesis-related genes was evaluated. The mRNA levels of *Pparg* and *Cebpa* were significantly higher in HFD-fed mice that received the EL fruit extract compared to those that did not receive the extract (Figure 3G,H). In contrast, the mRNA levels of *Hif1a* were lower in HFD-fed mice given the EL fruit extract compared to those not given the extract (Figure 3I).

### 3.4. EL Fruit Extract Attenuates HFD-Induced Inflammation and Oxidative Damage

**Figure 4:**
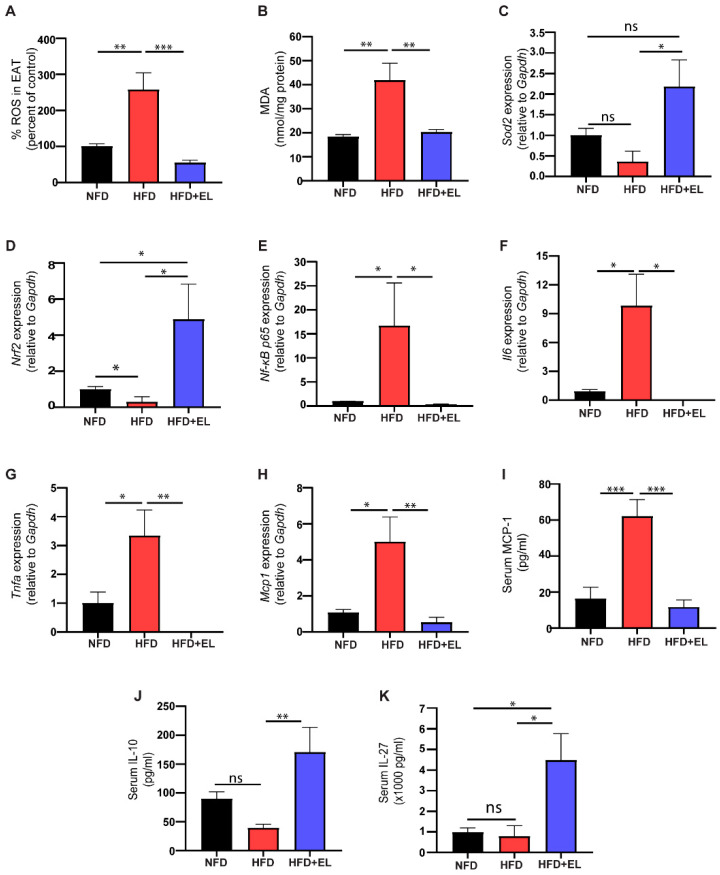
Effects of EL fruit extract on oxidative stress and HFD-induced inflammation. The levels of (**A**) reactive oxygen species (ROS) and (**B**) malondialdehyde (MDA). The relative mRNA expression of antioxidant-related genes (**C**) *Sod2*, (**D**) *Nrf2*, and inflammation-related genes (**E**) *Nf-κβ p65*, (**F**) *Il-6*, (**G**) *Tnf-α*, and (**H**) *Mcp-1*. The serum levels of (**I**) MCP-1, (**J**) IL10, and (**K**) IL27 (n = 6/group). The results are presented as the mean ± SEM, with statistical significance indicated as *, *p* < 0.05; **, *p* < 0.01; ***, *p* < 0.001.

To evaluate oxidative stress in adipocytes, ROS and MDA levels were measured. The results showed significantly higher levels of ROS and MDA in HFD-fed mice not given the EL fruit extract compared to those that received the extract (Figure 4A,B). Additionally, antioxidant genes, including *Nrf2* and *Sod2*, were upregulated in HFD-fed mice given the EL fruit extract compared to those not given the extract (Figure 4C,D). Inflammation-related genes, such as *NF-κB p65*, interleukin 6 (*Il6*), tumor necrosis factor-α (*Tnf*-α), and C-C motif chemokine ligand 2 (*Ccl2*/*Mcp1*), were significantly upregulated in HFD-fed mice not given the EL fruit extract compared to those receiving the extract (Figure 4E–H). These findings were consistent with the elevated serum levels of MCP1 (Figure 4I). Moreover, serum levels of IL10 and interleukin 27 (IL27) were higher in HFD-fed mice given the EL fruit extract than in those not given the extract (Figure 4J,K).

### 3.5. EL Fruit Extract Diminishes Macrophage Infiltration Surrounding the Adipocytes

**Figure 5:**
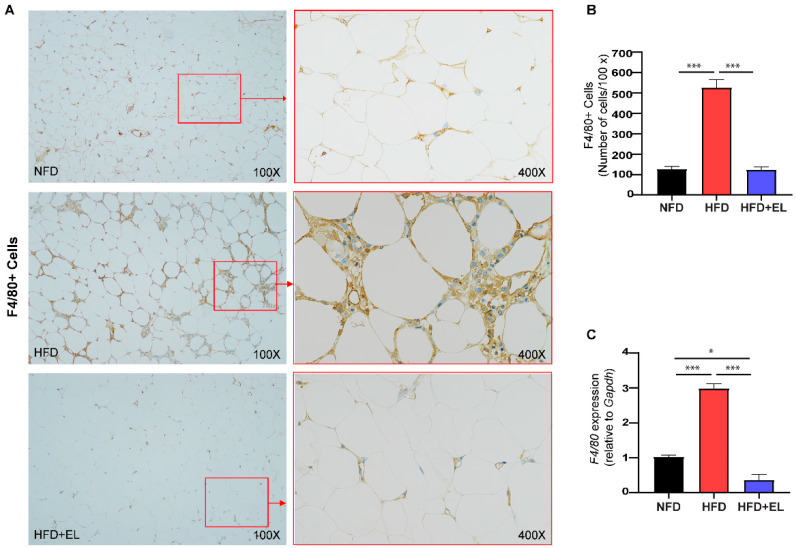
Effects of EL fruit extract on macrophage infiltration in EAT. (**A**) A representative immunohistochemical image of F4/80 (macrophages) at 100× and 400× magnification and (**B**) number of F4/80+ cells/100× magnification were demonstrated (n = 3–4/group). (**C**) The mRNA levels of *F4*/*80* were investigated (n = 6/group). The results are presented as the mean ± standard error SEM, with statistical significance indicated as *, *p* < 0.05; ***, *p* < 0.001.

Furthermore, immunohistochemical staining for the F4/80 protein on macrophage surfaces showed significantly higher expression in macrophages from HFD-fed mice not treated with the EL fruit extract compared to those from HFD-fed mice that were treated with the extract (Figure 5A,B). These findings are consistent with the increased mRNA levels of F4/80 in HFD-fed mice not treated with the EL fruit extract compared to those that received the extract (Figure 5C).

### 3.6. EL Fruit Extract Attenuates ROS and NO Production in Macrophages

**Figure 6:**
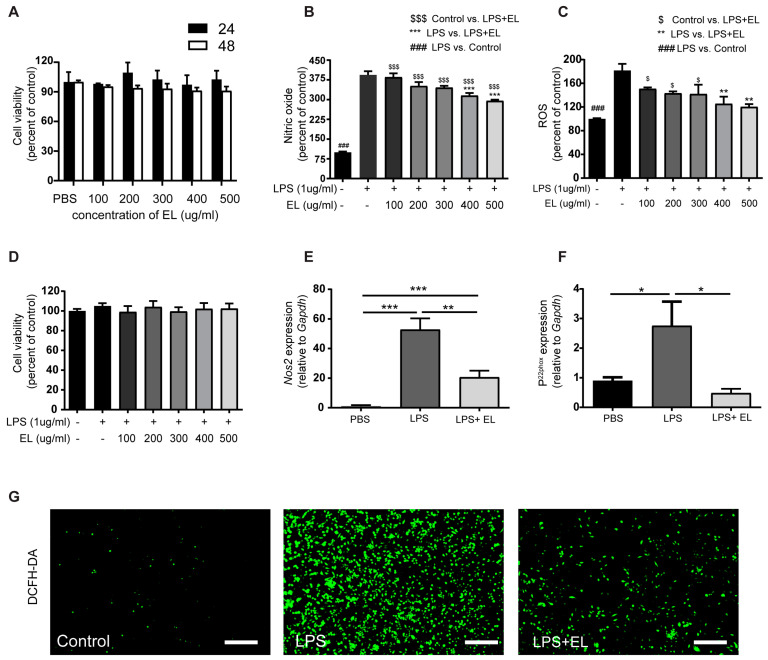
Effects of EL fruit extract on LPS-induced macrophages. (**A**) Cell viability was determined after treating macrophages with the EL fruit extract for 24 or 48 h using the MTS assay. The percentage of (**B**) NO and (**C**) ROS production. (**D**) The cytotoxic effect of various doses of the EL fruit extract in combination with LPS treatment. The mRNA levels of (**E**) *iN_OS_* and (**F**) *p22^phox^* (n = 4–6/group). (**G**) A representative image of ROS production using DCFH-DA staining observed under a fluorescent microscope at 100× magnification; scale bar = 10 µm (n = 3/group). The data are presented as the mean ± standard error SEM, with statistical significance indicated as *, *p* < 0.05; **, *p* < 0.01; ***, *p* < 0.001.

Next, the anti-inflammatory mechanism of the EL fruit extract in M1 macrophages surrounding adipocytes was investigated in vitro. Cell viability did not significantly differ between macrophages treated with various concentrations of the EL fruit extract and those that were not treated (Figure 6A). Additionally, treatment with 400–500 µg/mL of the EL fruit extract reduced NO and ROS production in activated macrophages compared to LPS-induced macrophages (Figure 6B,C). Cell viability also did not significantly differ between LPS-stimulated macrophages with or without EL fruit extract treatment and untreated macrophages (Figure 6D). Therefore, a maximum dose of 500 µg/mL of the EL fruit extract was used in subsequent experiments. Moreover, mRNA levels of inducible nitric oxide synthase (*Nos2*/*iNos*) and cytochrome b-245, alpha polypeptide (*Cyba*/*p^22phox^*) were downregulated in cells treated with the EL fruit extract compared to LPS-stimulated cells (Figure 6E,F). These findings were consistent with the reduced fluorescent signals from DCFH-DA staining in activated cells treated with the EL fruit extract (Figure 6G).

### 3.7. EL Fruit Extract Attenuates Inflammation by Reducing the Maturation and Cytokine Secretion of Activated Macrophages

**Figure 7:**
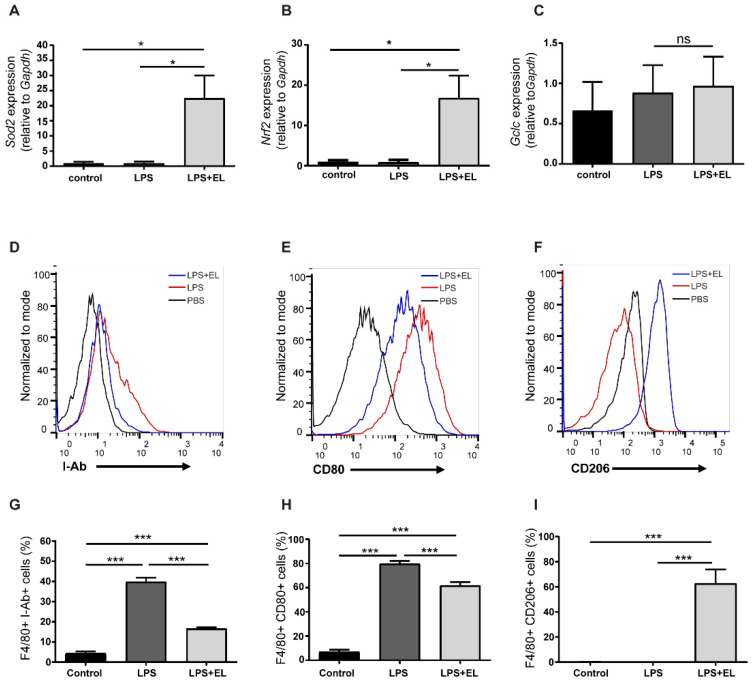
Effects of EL fruit extract on the maturation of activated macrophages. The mRNA levels of antioxidant-related genes (**A**) *Sod2*, (**B**) *Nrf2*, and (**C**) *Gclc*. Representative flow cytometry histogram of (**D**) F4/80+ I-Ab+ cells, (**E**) F4/80+ CD80+ cells, (**F**) F4/80+ CD206+ cells, and (**G**–**I**) the percentage were demonstrated. The data are presented as the mean ± standard error (SEM), with statistical significance exhibited as *, *p* < 0.05; ***, *p* < 0.001; ns, nonsignificant.

Treating activated macrophages with the EL fruit extract increased the mRNA levels of antioxidant-related genes, including *Sod2* and *Nrf2*, but did not affect the expression of glutamate-cysteine ligase, the catalytic subunit (*Gclc*) (Figure 7A–C). Additionally, EL fruit extract treatment reduced macrophage maturation by decreasing the percentages of surface CD80 and I-Ab MHC class II alloantigen, markers of M1-like macrophages, while significantly increasing CD206, an M2 macrophage surface marker, relative to LPS-induced macrophages (Figure 7D–I).

**Figure 8:**
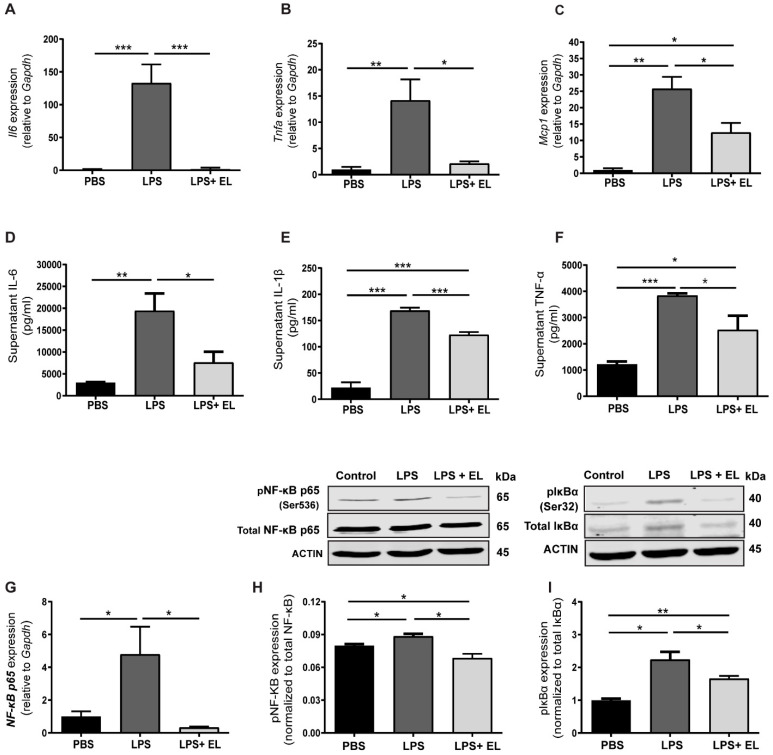
Effects of EL fruit extract on anti-inflammatory cytokine secretion. The cells and supernatant were collected after treating cells with the EL fruit extract with or without LPS for 24 h. The mRNA levels of (**A**) *Il-6*, (**B**) *Tnf-α*, and (**C**) *Mcp-1*. The protein levels of (**D**) IL-6, (**E**) IL-1β, and (**F**) TNF-α (n = 4–6/group). (**G**) The mRNA levels of *Nf-κB p65*. Western blot analysis of the protein levels of (**H**) total and phosphorylated (Ser536) NF-κB p65 and (**I**) total and phosphorylated (Ser32) IκBα in macrophages treated with the EL fruit extract with or without LPS for 60 min (n = 3–5/group). The results are presented as the mean ± standard error (SEM), with statistical significance exhibited as *, *p* < 0.05; **, *p* < 0.01; ***, *p* < 0.001.

Next, inflammatory cytokine levels were assessed. LPS-induced macrophages showed significantly higher mRNA levels of *Il6*, *Tnf-α*, and *Mcp1* (Figure 8A–C), which corresponded with elevated IL-6, TNF-α, and interleukin 1β (IL-1β) levels in the culture medium of LPS-induced macrophages compared to those treated with the EL fruit extract (Figure 8D–F). Both mRNA and protein levels of NF-κB p65 were significantly higher in LPS-induced macrophages than in those treated with the EL fruit extract (Figure 8G,H). Furthermore, phosphorylation of IκBα at Ser32 was reduced in activated macrophages treated with the EL fruit extract compared to untreated cells (Figure 8I).

### 3.8. EL Fruit Extract Attenuates LPS-Induced Macrophages by Reducing the Energy-Producing Pathways

**Figure 9:**
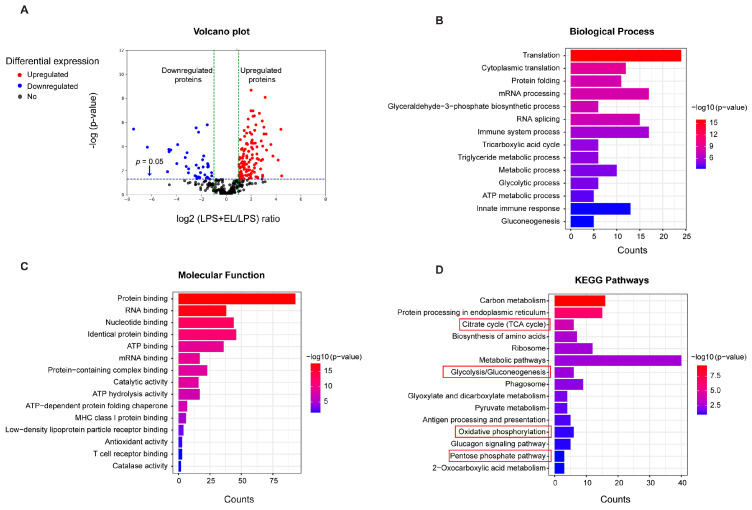
Proteomics analysis of activated macrophages treated with EL fruit extract. (**A**) Abundance of 306 differentially expressed proteins indicated in volcano plot between the LPS and LPS + EL groups (n = 3/group). (**B**) Biological process, (**C**) molecular function, and (**D**) KEGG annotation chart showing the Kyoto Encyclopedia of Genes and Genomes pathways of the 306 proteins differentially expressed between the LPS + EL and LPS groups. The red box indicates energy-producing pathways.

Proteomic analysis was conducted to gain a deeper understanding of how treatment with the EL fruit extract affects LPS-activated macrophages. This analysis identified 306 proteins that were differentially expressed between activated macrophages treated with LPS and those treated with both LPS and the EL fruit extract, as shown in the volcano plot (Figure 9A). The differentially expressed proteins were further analyzed and categorized according to their biological processes, molecular functions, and pathways (Figure 9B–D). Notably, several proteins involved in glycolysis and the tricarboxylic acid (TCA) cycle were found to be upregulated or downregulated in response to LPS activation (Supplementary Figure S1).

**Figure 10:**
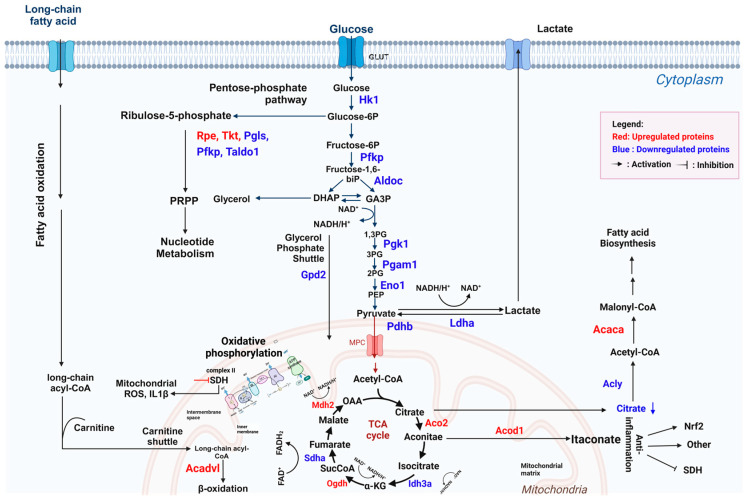
Schematic illustration of up- and downregulated proteins in glycolysis and TCA cycle from the proteomics analysis. Created with BioRender.com (accessed on 19 November 2024).

A number of downregulated proteins (highlighted in blue, Figure 10) were associated with (i) glycolysis, such as hexokinase 1 (HK1), a key enzyme in the rate-limiting step; (ii) the TCA cycle, including isocitrate dehydrogenase (IDH3A) and succinate dehydrogenase (SDHA); and (iii) fatty acid biosynthesis, such as ATP citrate lyase (ACLY). On the other hand, treatment with the EL fruit extract upregulated proteins involved in (i) fatty acid β-oxidation, including mitochondrial acyl-CoA dehydrogenase (ACADVL), and (ii) the TCA cycle, such as mitochondrial aconitase 2 (ACO2) and aconitate decarboxylase 1 (ACOD1). Data on the 154 upregulated and 152 downregulated proteins between the two groups are presented in Supplementary Table S2.

## 4. Discussion

Obesity-related inflammation and oxidative stress are key factors contributing to various metabolic diseases [2,23]. This study investigated the anti-obesity effects of an aqueous EL fruit extract and its potential mechanisms in reducing lipotoxicity-induced inflammatory and oxidative stress in WAT and macrophages. To minimize age-related confounding effects on neuroinflammation, immune responses, and metabolism [24,25], five-week-old mice were fed a HFD with or without the EL fruit extract. Our findings show that the administration of the EL fruit extract to HFD-fed mice significantly reduced BW, starting in week 5 and continuing through week 10, despite similar food and energy intake between the groups. It is widely recognized that excessive energy intake results in the accumulation of TGs in adipose tissue, leading to obesity [26]. This suggests that the EL fruit extract does not affect energy balance through hormonal or neurological mechanisms that alter food intake [27] but may reduce lipid accumulation in the adipose tissue. Specifically, the extract lowered total cholesterol, TGs, and FFAs while reducing the size of EAT. These effects are likely attributed to the total flavonoids [28] and polyphenols [29] in the EL fruit extract, which reduce lipid storage in adipocytes. Furthermore, the EL fruit extract reduced the number of CLSs in EAT and lowered *F4/80* expression, indicating reduced macrophage infiltration compared to HFD-fed mice not receiving the extract. These results are consistent with previous studies [30,31]. Additionally, oxidative stress was reduced in the EAT of HFD-fed mice treated with the EL fruit extract, as indicated by lower levels of MDA and ROS, alongside increased expression of the transcription factor *Nrf2* and its downstream superoxide dismutase 2 (*Sod2*), consistent with previous reports [32]. These antioxidant factors and enzymes play a critical role in scavenging lipid peroxides and preventing free radical propagation [9,33]. Notably, the EL fruit extract also increased the expression of the adipogenic transcription factors *Cebpa* and *Pparg* in EAT, suggesting a potential enhancement of adipogenesis in HFD-fed mice [34].

Our results are consistent with studies on plant extracts, such as those from *Artemisia* [35], *Mangifera indica* [36], and *Umbelliferae* [37], which also show anti-obesity effects. During obesity, adipocytes are enlarged due to hypertrophy (an increase in the size of existing adipocytes) rather than hyperplasia (an increase in their number), resulting from impaired adipogenesis (a reduction in the formation of new adipocytes) [1,38] and induced excessive cell death, which may exacerbate inflammation [39]. This enlargement can lead to local hypoxia, further exacerbating inflammation, adipocyte dysfunction, and insulin resistance by activating the oxygen-sensitive transcription factor HIF1A [6]. HIF-1 also inhibits adipogenesis by suppressing *Pparg* and *Cebpa* [40]. The EL fruit extract prevents the HFD-induced downregulation of *Hif1a* expression, implying that it may attenuate the vicious cycle of adipose tissue dysfunction by inhibiting HIF1A and enhancing adipogenic transcription factors. Elevated levels of inflammatory cytokines, such as TNF-α and MCP-1 [41], in inflamed adipose tissue are secreted by both macrophages and dysfunctional adipocytes [42,43]. Furthermore, in an obese environment, dysfunctional adipose tissue and leukocytes can produce MCP-1, which recruits inflammatory cells, especially macrophages, to the adipose tissue, ultimately inducing inflammatory, oxidative stress, and insulin-resistant states [44]. Our data suggest that the bioactive compounds in the EL fruit extract, including quercetin [15], catechin [45], and rutin [46], may stimulate adipogenesis by activating *Cebpa* and *Pparg*, improving adipocyte function in obese mice. Moreover, the activation of PPARG has been shown to reduce macrophage infiltration, adipocyte hypertrophy, and lipid accumulation in adipose tissue [47].

Moreover, the EL fruit extract increased *Pparg* expression (Figure 3G), which may inhibit the translocation of the PPARG/NF-κB complex to the nucleus, thereby shifting macrophages from a pro-inflammatory M1 phenotype, typically found in EAT, to an anti-inflammatory M2 phenotype [48]. This transition reduces the expression of pro-inflammatory cytokines, including TNF-α, IL-6, IL-1β, and MCP-1, while increasing IL-10 levels [49]. Additionally, elevated IL-27 levels may help prevent obesity by promoting uncoupling protein 1 (UCP1) production, enhancing adipocyte thermogenesis and energy expenditure in HFD-fed mice [50]. Increased TNF-α in HFD-fed mice binds to TNFR1 and TNFR2 receptors, activating NF-κB signaling and reducing AMP-activated protein kinase (AMPK) activity, which inhibits lipolysis and promotes lipogenesis in adipose tissue [39,51] Our data suggest that the EL fruit extract alleviates lipid accumulation in adipose tissue by inhibiting TNF-α/NF-κB signaling, thereby enhancing lipid catabolism through TNF-α/NF-κB-dependent AMPK pathways, as previously reported [39,51].

Macrophages are the primary immune cells infiltrating adipose tissue in obesity, often surrounding dead adipocytes [23,42]. To better understand how the EL fruit extract attenuates macrophage activation, we investigated LPS-induced macrophages in vitro. Treatment with the EL fruit extract reduced oxidative stress in LPS-activated macrophages by inhibiting *p^22phox^* signaling and promoted a shift from the M1 to M2 macrophage phenotype. This effect may be due to the polyphenols and flavonoids in the EL fruit extract, which reduce IκBα phosphorylation and inhibit NF-κB p65 activation. As a result, macrophages exhibit decreased expression of CD80, MHC-II, and iNOS, while CD206, a marker of M2-like macrophages, is upregulated [18,52].

Previous studies have demonstrated that LPS-activated macrophages enhance glycolysis, the TCA cycle, and the pentose phosphate pathway to support energy production in M1-like macrophages [53]. Our proteomic data show that treatment with the EL fruit extract downregulated proteins involved in glycolysis, the TCA cycle, and the pentose phosphate pathway in LPS-activated macrophages. These results suggest that the EL extract decreases energy production and the cytokine secretion-dependent glycolysis pathway in LPS-induced M1 macrophage activation [54]. Additionally, we observed higher expression of Acod1 in macrophages treated with both LPS and the EL fruit extract compared to LPS alone. This increased Acod1 expression may lead to elevated levels of itaconate, which promotes anti-inflammatory effects by activating NRF2 [55] and reducing mitochondrial ROS and cytokine production through the inhibition of succinate dehydrogenase (SDH)-mediated oxidative stress [56].

Phytochemical screening of the EL fruit extract revealed the presence of phenolic compounds, flavonoids, and their subtypes, including quercetin, reserpine, rutin, and catechin, consistent with previous studies [15,45,46]. LC-MS analysis identified additional bioactive compounds such as kaempferol, naringenin, stigmasterol, elaeagnoside F, and angustifolinoid. Quercetin, rutin, catechin, kaempferol, and naringenin are well-known for their anti-obesity, anti-fat accumulation, and anti-dyslipidemic effects in metabolic tissues like adipose and liver tissues in animal models of metabolic syndrome [57,58,59]. Quercetin, kaempferol, naringenin, and stigmasterol also exhibit anti-inflammatory and antioxidant properties by inhibiting inflammatory cell infiltration, suppressing NF-κB activation, reducing pro-inflammatory cytokine expression, and/or activating NRF2 and antioxidant enzymes in animal models [58,60,61].

Together, these findings suggest that the bioactive compounds in the EL fruit extract may work directly or synergistically to combat obesity, inflammation, and ROS production. Thus, aqueous EL fruit extract presents therapeutic potential for addressing obesity-associated inflammation and oxidative stress, particularly through macrophage modulation. Further studies are needed to explore the specific roles of key phytochemicals in the extract and their individual biopharmacological effects.

## 5. Conclusions

This study demonstrates the effects of an EL fruit extract, rich in phenolic subclasses and other bioactive compounds, on obesity-associated inflammation in macrophages. Our findings reveal multiple mechanisms through which the extract works, including the enhancement of CEBPA and PPARG expression, along with the suppression of HIF1A. These actions help improve adipocyte function and inhibit M1-like macrophage polarization and energy-producing pathways in activated macrophages. Additionally, the EL fruit extract enhances antioxidant protection in both mouse adipose tissue and macrophages. These results suggest that the EL fruit extract could be beneficial in attenuating obesity-related mechanisms, particularly metabolic dysregulation, immunomodulation, and oxidative damage. However, our study has some limitations, including the lack of data on the long-term effects of the EL fruit extract and its specific phytochemicals on female obesity and adipocyte culture. Further comprehensive studies are needed to address these gaps.

## Figures and Tables

**Table 1:** The phytochemical contents and antioxidant activities of aqueous EL fruit extract.

**Phytochemical Contents**	**Amount**
Total phenolic content (mg of GAE/g)	13.88 ± 0.41
Total flavonoid content (mg of catechin/g)	2.79 ± 0.10
**Antioxidant activity**	**IC_50_ values (µg/mL)**
DPPH of EL	289.96 ± 18.40
DPPH of Trolox	3.45 ± 0.28
ABTS of EL	151.30 ± 5.07
ABTS of Trolox	3.27 ± 0.07

The total phenolic content (TPC) and total flavonoid content (TFC) of the aqueous EL fruit extract were determined using colorimetric methods, as described earlier. The results are presented as the mean ± standard deviation (SD) (n = 3/group), with TPC expressed as gallic acid equivalents (GAE). Trolox was used as the positive control for antioxidant activity.

**Table 2:** Bioactive contents of the aqueous EL fruit extract determined using LC-MS/MS.

RT (min)	Mass	*m*/*z*	Identification	Chemical Formula
1.721	98.0371	143.0354	Angelica lactone	C_5_H_6_O_2_
1.721	126.0316	125.0244	Phloroglucinol	C_6_H_12_O_6_
1.759	304.0952	349.094	5-hydroxy-7,3′,4′-trimethoxyflavone	C_16_H_16_O_6_
1.759	286.0481	331.0459	Kaempferol	C_15_H_10_O_6_
7.157	302.0411	320.0751	Quercetin	C_15_H_10_O_7_
7.793	120.0579	121.0667	Phenyl acetaldehyde	C_8_H_8_O
8.168	290.0779	335.0765	Catechin	C_15_H_14_O_6_
10.604	386.1939	431.193	Roseoside	C_19_H_30_O_8_
16.213	204.116	263.1299	Isobutyl cinnamate	C_13_H_16_O_2_
16.447	168.0416	167.0343	Elaeagpyrone	C_8_H_8_O_6_
16.528	802.1948	801.1886	Isorhamnetin 3-0-{6-O-E-feruloyl)-β-d glucopyranosyl-(1-n)-β-d-galactopyranoside	C_37_H_38_O_20_
16.684	448.0991	447.0927	Naringenin 7-0-P-D-glucopyranoside	C_21_H_20_O_11_
17.009	128.0838	187.0977	Cyclohexane carboxylic acid	C_7_H_12_O_2_
17.513	594.1363	593.129	Angustifolinoid A	C_30_H_26_O_13_
17.943	176.0836	221.0818	Ethyl cinnamate	C_11_H_12_O_2_
17.943	162.0679	221.0818	Methyl cinnamate	C_10_H_10_O_2_
18.472	164.0835	223.0974	Phenylethyl acetate	C_10_H_12_O_2_
19.675	492.1265	491.119	5, 3′-dihydroxy-7,4′-dimethoxyflavone	C_23_H_24_O_12_
19.675	432.1052	491.1187	Vitexin	C_21_H_20_O_10_
19.675	150.0677	209.0811	2-hydroxy-5-methylacetophenone	C_9_H_10_O_2_
20.204	190.0989	249.1127	Isopropyl cinnamate	C_12_H_14_O_2_
20.204	204.1146	249.1127	Isobutyt cinnamate	C_13_H_16_O_2_
21.094	412.369	435.3523	Stigmasterol	C_29_H_48_O
21.434	608.2757	609.2837	Reserpine	C_33_H_40_N_2_O_9_
21.845	212.214	257.212	Tridecanoic acid, methyl ester	C_14_H_28_O
23.965	610.1508	633.1419	Rutin	C_27_H_30_O_16_
24.298	978.2636	1001.255	Elaeagnoside F	C_44_H_50_O_25_
24.355	576.4369	621.4357	Dausosterol	C_35_H_60_O_6_
26.704	594.139	617.1279	Angustifolinoid A	C_30_H_26_O_13_

LC-MS/MS was used to characterize the bioactive compounds in the aqueous EL fruit extract.

## Data Availability

The data presented in this study are available in the article. The mass spectrometry proteomics data are available via ProteomeXchange with the identifier PXD046383.

## Supplementary Materials

The following supporting information can be downloaded at: https://www.mdpi.com/article/10.3390/antiox13121485/s1: Table S1: IDs for each TaqMan gene expression assay. Table S2: Lists of up- and down-regulated proteins. Figure S1: lists of up- and downregulated proteins.
