# Supplementary material for: Elaeagnus latifolia Fruit Extract Ameliorates High-Fat Diet-Induced Obesity in Mice and Alleviates Macrophage-Induced Inflammation in Adipocytes In Vitro
Source: Antioxidants (Basel). 2024 Dec 5;13(12):1485. doi: 10.3390/antiox13121485 (PMC11673262; doi:10.3390/antiox13121485)
Supplement: Supplementary file 1 [file antioxidants-13-01485-s001.zip › antioxidants-3308656-supplementary.pdf]

Supplementary Figure S1

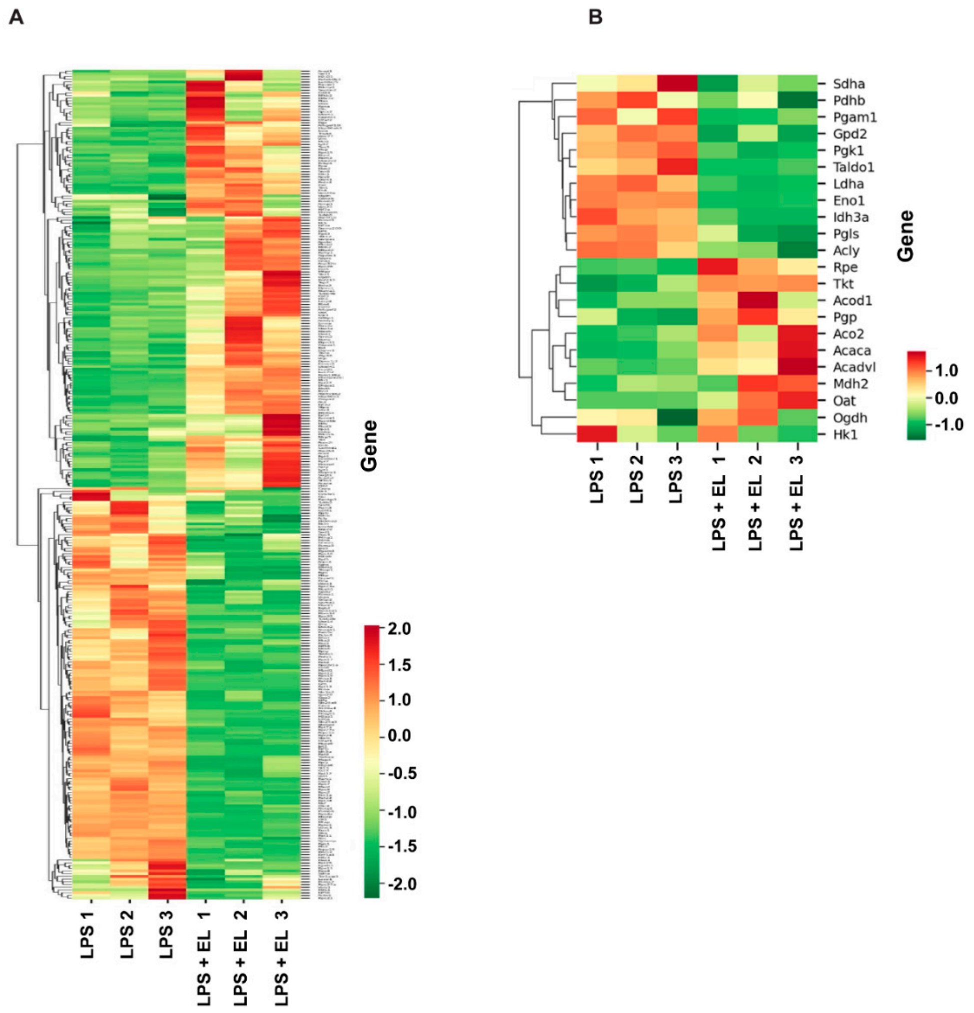

Supplementary Figure S1. Proteomics analysis of activated macrophages treated with the EL fruit extract. (A) The abundances of 306 differentially expressed proteins were normalized to calculate z-scores. The z-scores of protein abundance were clustered based on the mean of z-scores for the LPS and LPS+EL groups (n = 3/group). (B)The abundances of 23 proteins relevant to glucose metabolism (n = 3/group). Red and green denote up- and down-regulation, respectively. The color gradient indicates the z-score calculated from protein abundance levels. The first three columns are the protein abundance from three replicates of LPS treatment. The last three columns represent the protein abundance from three replicates of EL fruit extract treatment in LPS-activated macrophages.

Supplementary Table S1

IDs for each TaqMan gene expression assay.

Gene symbols	Assay IDs
PPARγ	Mm01184322_m1
C/EBPα	Mm00514283_s1
HIF-1α	Mm00468869_m1
NF-κB p65	Mm00501346_m1
IL-6	Mm00446190_m1
TNF-α	Mm00443258_m1
MCP-1	Mm00441242_m1
F4/80	Mm00802529_m1
Nrf2	Mm00477784_m1
Sod2	Mm01313000_m1
GCLC	Mm00802655_m1
GAPDH	Mm99999915_g1

Supplementary Table S2

Lists of up- and down-regulated proteins

Accession	Gene name	Description	LPS+EL/LPS	P-Value
P55097	Ctsk	Cathepsin K	4.46	0.0273
P35235	Ptpn11	Tyrosine-protein phosphatase non-receptor type 11	4.40	4E-06
P29758	Oat	Ornithine aminotransferase, mitochondrial	4.18	0.0013
Q9Z2G6	Sel1l	Protein sel-1 homolog 1	3.76	0.0006
P62960	Ybx1	Y-box-binding protein 1	3.59	8E-05
E9Q7G0	Numa1	Nuclear mitotic apparatus protein 1	3.26	0.0001
Q61205	Pafah1b3	Platelet-activating factor acetylhydrolase IB subunit alpha1	3.21	9E-06
Q99J09	Wdr77	Methylosome protein WDR77	3.14	1E-08
Q8BIG7	Comtd1	Catechol O-methyltransferase domain-containing protein 1	3.03	8E-06
P63037	Dnaja1	DnaJ homolog subfamily A member 1	3.03	2E-06
Q9Z0M5	Lipa	Lysosomal acid lipase/cholesteryl ester hydrolase	2.94	9E-05
Q3U0V2	Tradd	Tumor necrosis factor receptor type 1-associated DEATH domain protein	2.93	0.0011
P70195	Psmb7	Proteasome subunit beta type-7	2.93	0.0317
Q9CSU0	Rprd1b	Regulation of nuclear pre-mRNA domain-containing protein 1B	2.93	0.0236
Q9CR98	Fam136a	Protein FAM136A	2.90	8E-07
Q8BTW3	Exosc6	Exosome complex component MTR3	2.81	3E-05
Q80TM9	Nisch	Nischarin	2.80	0.0014
P97808	Fxyd5	FXYD domain-containing ion transport regulator 5	2.76	0.0074
Q9CWV6	Prkrip1	PRKR-interacting protein 1	2.67	2E-07
Q3U5Q7	Cmpk2	UMP-CMP kinase 2, mitochondrial	2.60	0.0011
Q8K1B8	Fermt3	Fermitin family homolog 3	2.55	0.0011
Q9CYN2	Spcs2	Signal peptidase complex subunit 2	2.55	0.0432
Q9DCW4	Etfb	Electron transfer flavoprotein subunit beta	2.52	0.0343
Q9CQ22	Lamtor1	Ragulator complex protein LAMTOR1	2.49	0.001

P70444	Bid	BH3-interacting domain death agonist	2.47	0.0001
P62843	Rps15	Small ribosomal subunit protein uS19	2.47	0.0004
Q9CR50	Rchy1	RING finger and CHY zinc finger domain-containing protein 1	2.40	0.0025
Q9R0E1	Plod3	Multifunctional procollagen lysine hydroxylase and glycosyltransferase LH3	2.39	0.0324
P40336	Vps26a	Vacuolar protein sorting-associated protein 26A	2.39	1E-06
P36371	Tap2	Antigen peptide transporter 2	2.34	5E-05
O08807	Prdx4	Peroxiredoxin-4	2.30	4E-06
O70475	Ugdh	UDP-glucose 6-dehydrogenase	2.27	0.0058
P61358	Rpl27	Large ribosomal subunit protein eL27	2.26	2E-07
P24668	M6pr	Cation-dependent mannose-6-phosphate receptor	2.26	0.0129
Q7TQH0	Atxn2l	Ataxin-2-like protein	2.26	0.0033
Q9Z1Q5	Clic1	Chloride intracellular channel protein 1	2.17	6E-05
O70194	Eif3d	Eukaryotic translation initiation factor 3 subunit D	2.16	8E-08
O88487	Dync1i2	Cytoplasmic dynein 1 intermediate chain 2	2.15	4E-07
Q5SWU9	Acaca	Acetyl-CoA carboxylase 1	2.13	0.0002
Q9D2M8	Ube2v2	Ubiquitin-conjugating enzyme E2 variant 2	2.12	0.0106
Q9D2V5	Aar2	Protein AAR2 homolog	2.10	3E-05
Q9CX34	Sugt1	Protein SGT1 homolog	2.09	0.0005
P62858	Rps28	Small ribosomal subunit protein eS28	2.05	0.002
Q8CIV8	Tbce	Tubulin-specific chaperone E	2.04	0.0029
A2AN08	Ubr4	E3 ubiquitin-protein ligase UBR4	2.03	6E-05
Q9CQT1	Mri1	Methylthioribose-1-phosphate isomerase	2.03	1E-05
Q9EST5	Anp32b	Acidic leucine-rich nuclear phosphoprotein 32 family member B	2.03	4E-05
P08905	Lyz2	Lysozyme C-2	2.03	0.0001
O08810	Eftud2	116 kDa U5 small nuclear ribonucleoprotein component	2.01	0.0165
Q62167	Ddx3x	ATP-dependent RNA helicase DDX3X	1.99	0.0425
Q924C1	Xpo5	Exportin-5	1.99	2E-09
Q8BML9	Qars1	Glutamine--tRNA ligase	1.99	1E-07
Q922Y1	Ubxn1	UBX domain-containing protein 1	1.97	0.0211
Q9CW46	Raver1	Ribonucleoprotein PTB-binding 1	1.95	0.0344
G5E829	Atp2b1	Plasma membrane calcium-transporting ATPase 1	1.92	6E-06
Q69ZK6	Jmjd1c	Probable JmjC domain-containing histone demethylation protein 2C	1.91	0.0012
P15379	Cd44	CD44 antigen	1.91	0.0381
Q8R081	Hnrnpl	Heterogeneous nuclear ribonucleoprotein L	1.89	0.0087
P11928	Oas1a	2'-5'-oligoadenylate synthase 1A	1.84	0.0213
P28740	Kif2a	Kinesin-like protein KIF2A	1.83	0.045
Q6P8X1	Snx6	Sorting nexin-6	1.82	0.0088
Q810B6	Ankfy1	Rabankyrin-5	1.82	0.0003
P97855	G3bp1	Ras GTPase-activating protein-binding protein 1	1.81	0.0047
P50544	Acadvl	Very long-chain specific acyl-CoA dehydrogenase, mitochondrial	1.81	0.002
Q61166	Mapre1	Microtubule-associated protein RP/EB family member 1	1.79	0.0002
O88842	Fgd3	FYVE, RhoGEF and PH domain-containing protein 3	1.78	0.0222
P58058	Nadk	NAD kinase	1.77	4E-07
E9Q555	Rnf213	E3 ubiquitin-protein ligase RNF213	1.75	0.0081
P98078	Dab2	Disabled homolog 2	1.74	0.0093
O88569	Hnrnpa2b1	Heterogeneous nuclear ribonucleoproteins A2/B1	1.73	1E-06

Q9DCH4	Eif3f	Eukaryotic translation initiation factor 3 subunit F	1.73	0.0098
O89086	Rbm3	RNA-binding protein 3	1.72	7E-05
P53996	Cnbp	CCHC-type zinc finger nucleic acid binding protein	1.68	0.0266
Q9EQK5	Mvp	Major vault protein	1.67	0.0097
Q8VEE0	Rpe	Ribulose-phosphate 3-epimerase	1.67	0.0006
Q91ZX7	Lrp1	Prolow-density lipoprotein receptor-related protein 1	1.66	0.0009
Q99PL5	Rrbp1	Ribosome-binding protein 1	1.64	5E-05
P14206	Rpsa	Small ribosomal subunit protein uS2	1.64	0.0084
P08113	Hsp90b1	Endoplasmin	1.63	0.0029
Q9WTP6	Ak2	Adenylate kinase 2, mitochondrial	1.63	8E-06
P97287	Mcl1	Induced myeloid leukemia cell differentiation protein Mcl-1 homolog	1.61	6E-05
P57784	Snrpa1	U2 small nuclear ribonucleoprotein A'	1.60	0.0123
P01900	H2-D1	H-2 class I histocompatibility antigen, D-D alpha chain	1.55	0.0238
P00405	Mtco2	Cytochrome c oxidase subunit 2	1.53	0.0002
Q99NB9	Sf3b1	Splicing factor 3B subunit 1	1.50	0.0032
Q7TNC4	Luc7l2	Putative RNA-binding protein Luc7-like 2	1.48	1E-05
Q8BTI8	Srrm2	Serine/arginine repetitive matrix protein 2	1.48	0.0018
Q8R317	Ubqln1	Ubiquilin-1	1.46	0.0053
P62814	Atp6v1b2	V-type proton ATPase subunit B, brain isoform	1.45	4E-06
O88456	Capns1	Calpain small subunit 1	1.44	0.0071
Q8BIQ5	Cstf2	Cleavage stimulation factor subunit 2	1.42	0.0079
Q8CG72	Adprs	ADP-ribosylhydrolase ARH3	1.42	0.0457
Q9CZ13	Uqcrc1	Cytochrome b-c1 complex subunit 1, mitochondrial	1.41	0.0002
Q60875	Arhgef2	Rho guanine nucleotide exchange factor 2	1.39	0.0004
P24270	Cat	Catalase	1.38	0.0022
Q9R0P3	Esd	S-formylglutathione hydrolase	1.36	0.0005
P48678	Lmna	Prelamin-A/C	1.35	0.0036
P80316	Cct5	T-complex protein 1 subunit epsilon	1.32	0.0264
P47753	Capza1	F-actin-capping protein subunit alpha-1	1.32	0.001
Q99K48	Nono	Non-POU domain-containing octamer-binding protein	1.31	0.0016
Q8BGS2	Bola2	BolA-like protein 2	1.30	0.0045
P54987	Acod1	Cis-aconitate decarboxylase	1.30	0.0492
P70349	Hint1	Adenosine 5'-monophosphoramidase HINT1	1.30	0.0024
O54825	Bysl	Bystin	1.29	0.0001
P08249	Mdh2	Malate dehydrogenase, mitochondrial	1.25	0.04
A2AWP8	Arhgef10l	Rho guanine nucleotide exchange factor 10-like protein	1.25	0.0182
Q6R0H7	Gnas	Guanine nucleotide-binding protein G(s) subunit alpha isoforms XLas	1.25	0.0276
Q9D8E6	Rpl4	Large ribosomal subunit protein uL4	1.21	0.0024
Q80X90	Flnb	Filamin-B	1.21	0.0186
P68372	Tubb4b	Tubulin beta-4B chain	1.21	0.0002
P11499	Hsp90ab1	Heat shock protein HSP 90-beta	1.19	0.0051
Q5SUR0	Pfas	Phosphoribosylformylglycinamidine synthase	1.19	0.0117
O08795	Prkcsh	Glucosidase 2 subunit beta	1.15	0.0009
Q9D1L9	Lamtor5	Ragulator complex protein LAMTOR5	1.14	3E-05
Q9EQH3	Vps35	Vacuolar protein sorting-associated protein 35	1.13	0.0144
Q8BWW4	Larp4	La-related protein 4	1.11	0.0052
Q8VBT6	Apobr	Apolipoprotein B receptor	1.10	0.0425
Q99KI0	Aco2	Aconitate hydratase, mitochondrial	1.09	0.0143
P31230	Aimp1	Aminoacyl tRNA synthase complex-interacting multifunctional protein 1	1.08	0.0007

Q9D819	Ppa1	Inorganic pyrophosphatase	1.06	0.0011
Q8R4B8	Nlrp3	NACHT, LRR and PYD domains-containing protein 3	1.05	0.0069
P26369	U2af2	Splicing factor U2AF 65 kDa subunit	1.05	0.0265
P42208	Septin2	Septin-2	1.04	0.0285
O35375	Nrp2	Neuropilin-2	1.03	0.0031
P26039	Tln1	Talin-1	1.03	0.0046
P17897	Lyz1	Lysozyme C-1	1.02	0.0242
Q64337	Sqstm1	Sequestosome-1	0.99	0.0458
Q5SSZ5	Tns3	Tensin-3	0.99	0.0338
Q9WTK5	Nfkb2	Nuclear factor NF-kappa-B p100 subunit	0.99	0.0451
P22893	Zfp36	mRNA decay activator protein ZFP36	0.97	0.0001
P08103	Hck	Tyrosine-protein kinase HCK	0.92	0.0472
Q8CHP8	Pgp	Glycerol-3-phosphate phosphatase	0.90	0.0163
P21460	Cst3	Cystatin-C	0.88	0.0013
Q9QXS1	Plec	Plectin	0.87	0.0014
Q3TLH4	Prrc2c	Protein PRRC2C	0.87	0.0032
Q99P72	Rtn4	Reticulon-4	0.82	0.0259
P14148	Rpl7	Large ribosomal subunit protein uL30	0.78	0.0302
Q68FD5	Cltc	Clathrin heavy chain 1	0.76	0.0191
P20152	Vim	Vimentin	0.73	0.0055
O55135	Eif6	Eukaryotic translation initiation factor 6	0.71	0.0202
Q8VIJ6	Sfpq	Splicing factor, proline- and glutamine-rich	0.71	0.0056
Q8BJY1	Psmd5	26S proteasome non-ATPase regulatory subunit 5	0.65	0.0374
Q9JKR6	Hyou1	Hypoxia up-regulated protein 1	0.65	0.007
P40142	Tkt	Transketolase	0.62	0.004
Q6P4T2	Snrnp200	U5 small nuclear ribonucleoprotein 200 kDa helicase	0.62	0.0039
Q3UDE2	Ttll12	Tubulin--tyrosine ligase-like protein 12	0.59	0.0287
Q01853	Vcp	Transitional endoplasmic reticulum ATPase	0.56	0.0017
Q9D0E1	Hnrnpm	Heterogeneous nuclear ribonucleoprotein M	0.56	4E-07
P23116	Eif3a	Eukaryotic translation initiation factor 3 subunit A	0.51	0.0168
P01897	H2-L	H-2 class I histocompatibility antigen, L-D alpha chain	0.42	0.002
Q922F4	Tubb6	Tubulin beta-6 chain	0.42	9E-06
Q8BHN3	Ganab	Neutral alpha-glucosidase AB	0.31	0.0145
Q60597	Ogdh	2-oxoglutarate dehydrogenase complex component E1	0.27	0.0185
P56480	Atp5f1b	ATP synthase subunit beta, mitochondrial	0.05	0.0325
P17710	Hk1	Hexokinase-1	-0.23	0.0232
P24452	Capg	Macrophage-capping protein	-0.24	0.0064
Q8K2B3	Sdha	Succinate dehydrogenase [ubiquinone] flavoprotein subunit, mitochondrial	-0.47	0.0433
Q9Z1Q9	Vars1	Valine--tRNA ligase	-0.56	0.0429
Q9D051	Pdhb	Pyruvate dehydrogenase E1 component subunit beta, mitochondrial	-0.59	0.0035
Q8VI93	Oas3	2'-5'-oligoadenylate synthase 3	-0.62	0.0193
P57780	Actn4	Alpha-actinin-4	-0.65	0.009
Q61792	Lasp1	LIM and SH3 domain protein 1	-0.68	0.0032
P09103	P4hb	Protein disulfide-isomerase	-0.77	0.0028
Q8K124	Plekho2	Pleckstrin homology domain-containing family O member 2	-0.79	0.0045
P80313	Cct7	T-complex protein 1 subunit eta	-0.87	0.0016
P49722	Psma2	Proteasome subunit alpha type-2	-1.17	0.0286
Q8C3J5	Dock2	Dedicator of cytokinesis protein 2	-1.18	0.0149
Q9CWJ9	Atic	Bifunctional purine biosynthesis protein ATIC	-1.45	0.0046

P32921	Wars1	Tryptophan--tRNA ligase, cytoplasmic	-1.46	0.0437
P99024	Tubb5	Tubulin beta-5 chain	-1.46	0.0036
P08752	Gnai2	Guanine nucleotide-binding protein G(i) subunit alpha-2	-1.50	0.0028
Q922Q8	Lrrc59	Leucine-rich repeat-containing protein 59	-1.50	0.005
P08228	Sod1	Superoxide dismutase [Cu-Zn]	-1.54	2E-06
Q8BTM8	Flna	Filamin-A	-1.59	0.0415
Q91V92	Acly	ATP-citrate synthase	-1.73	0.0224
P09411	Pgk1	Phosphoglycerate kinase 1	-1.73	0.0014
Q8CIN4	Pak2	Serine/threonine-protein kinase PAK 2	-1.81	0.0006
P63017	Hspa8	Heat shock cognate 71 kDa protein	-1.86	0.0051
Q8R180	Ero1a	ERO1-like protein alpha	-1.91	0.0012
Q68FL6	Mars1	Methionine--tRNA ligase, cytoplasmic	-2.03	0.0373
P62983	Rps27a	Ubiquitin-ribosomal protein eS31 fusion protein	-2.08	0.0149
Q8C1A5	Thop1	Thimet oligopeptidase	-2.16	0.0425
P35979	Rpl12	Large ribosomal subunit protein uL11	-2.21	0.0062
O54734	Ddost	Dolichyl-diphosphooligosaccharide--protein glycosyltransferase 48 kDa subunit	-2.22	6E-06
Q9CQ60	Pgls	6-phosphogluconolactonase	-2.23	0.0339
P62242	Rps8	Small ribosomal subunit protein eS8	-2.37	0.0474
P38647	Hspa9	Stress-70 protein, mitochondrial	-2.41	0.0035
P16045	Lgals1	Galectin-1	-2.42	0.0308
Q3U0V1	Khsrp	Far upstream element-binding protein 2	-2.42	3E-06
Q60715	P4ha1	Prolyl 4-hydroxylase subunit alpha-1	-2.52	0.0267
Q9R1P4	Psma1	Proteasome subunit alpha type-1	-3.02	0.0071
P17742	Ppia	Peptidyl-prolyl cis-trans isomerase A	-3.04	0.015
P06151	Ldha	L-lactate dehydrogenase A chain	-3.16	0.0004
Q9DBJ1	Pgam1	Phosphoglycerate mutase 1	-3.22	0.0045
P10107	Anxa1	Annexin A1	-3.31	0.0008
Q922R8	Pdia6	Protein disulfide-isomerase A6	-3.90	0.0469
Q62465	Vat1	Synaptic vesicle membrane protein VAT-1 homolog	-3.90	0.0489
P28798	Grn	Progranulin	-3.92	7E-05
Q9JKP5	Mbnl1	Muscleblind-like protein 1	-4.00	0.0393
Q8C2Q3	Rbm14	RNA-binding protein 14	-4.02	0.0429
Q62159	Rhoc	Rho-related GTP-binding protein RhoC	-4.07	0.0369
Q9CY58	Serbp1	SERPINE1 mRNA-binding protein 1	-4.12	0.0413
P62754	Rps6	Small ribosomal subunit protein eS6	-4.17	0.03
Q9D883	U2af1	Splicing factor U2AF 35 kDa subunit	-4.23	0.0262
Q76MZ3	Ppp2r1a	Serine/threonine-protein phosphatase 2A 65 kDa regulatory subunit A alpha isoform	-4.25	0.0298
Q61768	Kif5b	Kinesin-1 heavy chain	-4.29	0.0239
P25444	Rps2	Small ribosomal subunit protein uS5	-4.43	0.0275
P62911	Rpl32	Large ribosomal subunit protein eL32	-4.47	0.0002
Q9CQR2	Rps21	Small ribosomal subunit protein eS21	-4.53	0.0391
P14115	Rpl27a	Large ribosomal subunit protein uL15	-4.55	0.0002
P17182	Eno1	Alpha-enolase	-4.61	0.0003
P10126	Eef1a1	Elongation factor 1-alpha 1	-4.61	0.0001
Q3TWW8	Srsf6	Serine/arginine-rich splicing factor 6	-4.67	0.01
P46460	Nsf	Vesicle-fusing ATPase	-4.72	0.0119
P42932	Cct8	T-complex protein 1 subunit theta	-4.77	0.029
P62702	Rps4x	Small ribosomal subunit protein eS4	-4.78	0.0086
Q9WV32	Arpc1b	Actin-related protein 2/3 complex subunit 1B	-4.83	0.01

Q64521	Gpd2	Glycerol-3-phosphate dehydrogenase, mitochondrial	-4.88	0.0498
P63325	Rps10	Small ribosomal subunit protein eS10	-4.89	0.0495
Q99LP6	Grpel1	GrpE protein homolog 1, mitochondrial	-4.89	0.0487
O35387	Hax1	HCLS1-associated protein X-1	-4.90	0.0477
Q9WUA3	Pfkp	ATP-dependent 6-phosphofructokinase, platelet type	-4.91	0.0473
Q8BKC5	Ipo5	Importin-5	-4.91	0.0466
Q921T2	Tor1aip1	Torsin-1A-interacting protein 1	-4.92	0.0459
P62751	Rpl23a	Large ribosomal subunit protein uL23	-4.92	0.0456
P27546	Map4	Microtubule-associated protein 4	-4.94	0.0442
P97384	Anxa11	Annexin A11	-4.95	0.0432
Q8BLF1	Nceh1	Neutral cholesterol ester hydrolase 1	-4.95	0.0432
Q8BKS9	Pum3	Pumilio homolog 3	-4.95	0.0432
Q9DB73	Cyb5r1	NADH-cytochrome b5 reductase 1	-4.97	0.0412
Q9WV55	Vapa	Vesicle-associated membrane protein-associated protein A	-4.97	0.0412
Q99L47	St13	Hsc70-interacting protein	-4.99	0.0393
Q6P5E6	Gga2	ADP-ribosylation factor-binding protein GGA2	-4.99	0.0387
Q9Z2U1	Psma5	Proteasome subunit alpha type-5	-5.00	0.0384
P40124	Cap1	Adenylyl cyclase-associated protein 1	-5.01	4E-05
P10852	Slc3a2	Amino acid transporter heavy chain SLC3A2	-5.05	0.0337
Q9D1C8	Vps28	Vacuolar protein sorting-associated protein 28 homolog	-5.07	0.0317
P62259	Ywhae	14-3-3 protein epsilon	-5.08	0.0309
Q9EQ61	Pes1	Pescadillo homolog	-5.09	0.0307
P47962	Rpl5	Large ribosomal subunit protein uL18	-5.09	0.015
Q8VCM8	Ncln	BOS complex subunit NCLN	-5.10	0.0299
P47791	Gsr	Glutathione reductase, mitochondrial	-5.12	0.0281
Q8BJU0	Sgta	Small glutamine-rich tetratricopeptide repeat-containing protein alpha	-5.12	0.0281
P35278	Rab5c	Ras-related protein Rab-5C	-5.12	0.0278
P59999	Arpc4	Actin-related protein 2/3 complex subunit 4	-5.13	0.0273
Q810D6	Grwd1	Glutamate-rich WD repeat-containing protein 1	-5.13	0.0271
Q9QYB1	Clic4	Chloride intracellular channel protein 4	-5.14	0.0264
Q9CT10	Ranbp3	Ran-binding protein 3	-5.16	0.0254
Q9CXW4	Rpl11	Large ribosomal subunit protein uL5	-5.16	0.0066
Q64324	Stxbp2	Syntaxin-binding protein 2	-5.17	0.0245
P07091	S100a4	Protein S100-A4	-5.17	0.0138
Q9QWV9	Ccnt1	Cyclin-T1	-5.18	0.0238
P62806	H4c1	Histone H4	-5.18	0.0022
Q99LC5	Etfa	Electron transfer flavoprotein subunit alpha, mitochondrial	-5.21	0.0221
P24369	Ppib	Peptidyl-prolyl cis-trans isomerase B	-5.21	0.0221
Q05D44	Eif5b	Eukaryotic translation initiation factor 5B	-5.24	0.0201
P63323	Rps12	Small ribosomal subunit protein eS12	-5.26	0.002
Q9CWF2	Tubb2b	Tubulin beta-2B chain	-5.27	0.0185
Q60692	Psmb6	Proteasome subunit beta type-6	-5.28	0.0178
P12970	Rpl7a	Large ribosomal subunit protein eL8	-5.28	0.0016
P62852	Rps25	Small ribosomal subunit protein eS25	-5.29	0.0174
P11835	Itgb2	Integrin beta-2	-5.29	0.0173
Q64433	Hspe1	10 kDa heat shock protein, mitochondrial	-5.29	0.0171
Q9R190	Mta2	Metastasis-associated protein MTA2	-5.29	0.0171
P68433	H3c1	Histone H3.1	-5.32	0.0157
Q9D0I9	Rars1	Arginine--tRNA ligase, cytoplasmic	-5.32	0.0156

Q9JMA1	Usp14	Ubiquitin carboxyl-terminal hydrolase 14	-5.36	0.014
P35564	Canx	Calnexin	-5.37	0.0134
P20060	Hexb	Beta-hexosaminidase subunit beta	-5.37	0.0132
P47955	Rplp1	Large ribosomal subunit protein P1	-5.38	0.013
Q99K51	Pls3	Plastin-3	-5.41	0.0117
Q8VEM8	Slc25a3	Solute carrier family 25 member 3	-5.42	0.0114
Q9D6R2	Idh3a	Isocitrate dehydrogenase [NAD] subunit alpha, mitochondrial	-5.44	0.0071
Q99L28	Rsl24d1	Probable ribosome biogenesis protein RLP24	-5.47	0.0095
Q9QZD9	Eif3i	Eukaryotic translation initiation factor 3 subunit I	-5.50	0.0085
Q7TMK9	Syncrip	Heterogeneous nuclear ribonucleoprotein Q	-5.51	0.0082
P62918	Rpl8	Large ribosomal subunit protein uL2	-5.51	0.0082
A2AR02	Ppig	Peptidyl-prolyl cis-trans isomerase G	-5.51	0.0081
P51881	Slc25a5	ADP/ATP translocase 2	-5.54	0.0073
P35700	Prdx1	Peroxiredoxin-1	-5.56	0.0016
Q60932	Vdac1	Voltage-dependent anion-selective channel protein 1	-5.57	0.0066
P97825	Jpt1	Jupiter microtubule associated homolog 1	-5.58	0.0062
P56212	Arpp19	cAMP-regulated phosphoprotein 19	-5.59	0.0061
P63158	Hmgb1	High mobility group protein B1	-5.70	0.0037
Q9CQ65	Mtap	S-methyl-5'-thioadenosine phosphorylase	-5.72	0.0034
Q9D1R9	Rpl34	Large ribosomal subunit protein eL34	-5.73	0.0033
P62830	Rpl23	Large ribosomal subunit protein uL14	-5.75	0.0029
Q9CXI5	Manf	Mesencephalic astrocyte-derived neurotrophic factor	-5.78	0.0026
P63276	Rps17	Small ribosomal subunit protein eS17	-5.81	0.0022
P0C0S6	H2az1	Histone H2A.Z	-5.84	0.0019
P61255	Rpl26	Large ribosomal subunit protein uL24	-5.85	0.0018
Q9CQW9	Ifitm3	Interferon-induced transmembrane protein 3	-5.87	0.0016
Q9QUH0	Glrx	Glutaredoxin-1	-5.89	0.0014
Q93092	Taldo1	Transaldolase	-5.97	0.0009
P54116	Stom	Stomatin	-5.97	0.0009
P17918	Pcna	Proliferating cell nuclear antigen	-6.01	0.0001
P57759	Erp29	Endoplasmic reticulum resident protein 29	-6.02	0.0006
Q8CBY8	Dctn4	Dynactin subunit 4	-6.06	0.0005
P18760	Cfl1	Cofilin-1	-6.08	0.0004
Q8BK64	Ahsa1	Activator of 90 kDa heat shock protein ATPase homolog 1	-6.09	0.0004
P62082	Rps7	Small ribosomal subunit protein eS7	-6.10	0.0004
Q8BP67	Rpl24	Large ribosomal subunit protein eL24	-6.10	0.0003
P62281	Rps11	Small ribosomal subunit protein uS17	-6.12	0.0003
Q9CZX8	Rps19	Small ribosomal subunit protein eS19	-6.17	6E-05
P18572	Bsg	Basigin	-6.20	0.0001
P47963	Rpl13	Large ribosomal subunit protein eL13	-6.22	4E-05
